# Breast cancer survivors’ participation in social activities five years after primary surgery – are there social inequalities?

**DOI:** 10.1186/s12885-025-15297-0

**Published:** 2025-11-27

**Authors:** Stefanie Sperlich, Siegfried Geyer, Sophia Holthausen-Markou, Tjoung-Won Park-Simon, Batoul Safieddine, Eranda Sahiti, Dorothee Noeres

**Affiliations:** 1 Department of Medical Sociology, Comprehensive Cancer Center, Hannover Medical School, Hannover, Germany; 2 Gynaecological Psychosomatics and Psychooncology, Clinic for Gynaecology and Obstetrics, Hannover Medical School, Hannover, Germany; 3 Clinic for Gynaecology and Obstetrics, Hannover Medical School, Hannover, Germany

**Keywords:** Social participation, Social inequalities, Social activities, Breast cancer, Long term care

## Abstract

**Background:**

To date, the social participation of breast cancer survivors has mainly been studied in terms of their return to work, while long-term studies on participation in social activities are rare. Against this backdrop, we analyze the influence of socioeconomic and sociodemographic factors on breast cancer survivors’ social activities on average five years following first surgery. Furthermore, we identify social subgroups that are most affected by low levels of social activities, taking long-term breast cancer-related complaints into account.

**Methods:**

The study population is based on a multicenter longitudinal study on return to work after breast cancer. We used data of a third follow-up study with *n* = 372 breast cancer survivors, corresponding to a response rate of 81.9% with regard to the first sample. Participation in social activities comprises the dimensions of ‘sociocultural participation’, ‘social participation in institutions’ and ‘social participation in the private sphere’. Logistic regression analyses and CHAID decision tree analyses were applied to analyze the influence of socioeconomic factors (school education, income and occupational position), sociodemographic factors (age, marital status, labor force participation and migration status) and the number of cancer-related complaints on social participation.

**Results:**

Five years after the primary surgery, younger survivors (≤ 61 years) with a high number of breast cancer-related complaints showed the greatest limitations in ‘social participation in the private sphere’. Marital status, labor force participation and migration status were of less importance in all the dimensions of social participation considered. By contrast, all socioeconomic factors proved to be significantly associated with sociocultural participation. In particular, women with a low household income with more than two breast cancer-related complaints were identified as a vulnerable group in terms of low sociocultural participation.

**Conclusions:**

We found evidence of social inequalities in participation in social activities among breast cancer survivors. Our results indicate that a comprehensive assessment of social participation and early intervention are required to prevent long-term limitations in social activities, especially in socially disadvantaged breast cancer survivors.

**Supplementary Information:**

The online version contains supplementary material available at 10.1186/s12885-025-15297-0.

## Background

Social participation can be defined as a person’s involvement in activities that provide interactions with others in community life and in important shared spaces [1, 2]. Social participation is the primary goal of rehabilitation, which aims to improve functional health and enables a return to normal life and society [3]. The importance of social participation is reflected in the International Classification of Functioning, Disability and Health (ICF), in which social participation is taken into account in the assessment of the consequences of a disease [4]. According to the ICF, ‘participation’ (code d9) refers to the involvement in social and civic life, e.g. by going to church, taking part in cultural activities, leisure activities or going on holiday.

Previous studies have shown that social participation improves self-efficacy in coping with disabilities and psychological resilience [5, 6]. Furthermore, findings indicate significant associations between social participation, well-being and health-related quality of life [7–9]. Glass et al. found that socially active older adults even had a lower risk of all-cause mortality than those who were less socially active. They concluded that social and productive activities provide equivalent survival benefits compared to fitness activities [10]. One possible mechanism is that social activities involve the adoption of meaningful social roles, which promotes feelings of self-efficacy and a sense of purpose in life, which in turn has been linked to health outcomes [11, 12]. In addition, social participation can contribute to social connectedness, i.e. to the feeling of belonging to a social relationship or network [13]. Being integrated into social networks has been shown to be a protective factor for survival [14, 15].

### Social participation of breast cancer patients

So far, social participation of breast cancer survivors has been analyzed mainly in terms of return to work [16–19], while studies focusing on daily social activities and involvement in life situations beyond employment are rare. Of the few studies, Zhu et al. analyzed social participation in young and middle-aged breast cancer patients six months after surgery. They found that overall social participation status was poor, particularly in social dimensions including social withdrawal and lack of collective activities [3]. Similarly, Fangel et al. [20] found that one year after breast cancer treatment, women experienced changes in functional abilities that negatively affected daily activities and social participation, leading to impaired quality of life. Sperlich et al. showed that breast cancer patients participated less in social life compared to the general population, particularly in terms of “sociocultural participation”, which includes visiting cafes, pubs or restaurants, going on short trips or attending cultural events. Mental health symptoms, pain and a history of mastectomy were the most important diagnosis-related issues associated with limitations in social participation [21]. The qualitative study by Nikolić et al. points into the same direction, showing that breast cancer patients found it more difficult to maintain their social activities as compared to everyday activities. The authors concluded that measures aimed at preventing social exclusion and at strengthening personal resources concerning social roles of women with breast cancer are of great importance [22]. Loubani et al. [23] assessed the retained activity levels of breast cancer survivors compared with pre-diagnosis activity levels in sociocultural, physical, and instrumental domains. They found higher participation restrictions for women two years post diagnosis compared with the situation five years later. Almost half of the women achieved significant changes in their meaningful activities, however, participation restrictions remained even five years following diagnosis. Beasley et al. [24] prospectively examined the role of social connectedness for survival after breast cancer diagnosis. They found that women who scored high on social connectedness as determined by the frequency of contacts with family and friends, attendance of religious services, and participation in community activities, had a lower risk of breast cancer mortality and of all-cause mortality.

### Social inequalities in social participation

People with low socioeconomic status are more likely to have restrictions in social participation. For example, Wilki et al. [25] found that interpersonal interaction and social activities in persons with lower socio-economic status are less likely to be “as and when you want it”. Similar, Bukov et al. [26] found that educational and occupational resources were positively related to the intensity of social participation in old age. The systematic reviews by Townsend et al. [27] and Mousa Garmabi et al. [28] also showed that low socioeconomic status is a barrier to social participation for older adults. In addition, other social factors are associated with restrictions on social participation, such as living without a partner [29, 30], having a migration background [31–33] and being not employed [34].

So far, research on social inequalities regarding social participation among breast cancer survivors is scarce, particular with regard to a long-term perspective. In one of the few studies on this topic, Zhu et al. found no significant influence of education or income level on the social participation of breast cancer patients [3]. Against this backdrop, we analyzed the influence of social factors on levels of participation in social activities on average five years following first surgery. Based on previous findings, we hypothesize that socially disadvantaged breast cancer survivors are more restricted in their social participation than socially better-off survivors. Furthermore, we assume that living without a partner, being not employed and having a migration background is associated with limited social participation. To test these hypotheses, we first determine the influence of socioeconomic and sociodemographic factors on various dimensions of social participation. We also determine the relevance of diagnosis-related complaints and investigate whether the relationship between social factors and social participation is mediated by diagnosis-related complaints. Finally, we analyze what subgroups of breast cancer survivors are affected most by low levels of social participation.

## Methods

### Data source

#### Breast cancer patients

The study population is based on a multicenter longitudinal study, initially focused on return to work after breast cancer in Lower Saxony, Germany. The recruitment took place in 10 certified breast cancer centers, and at one gynecologist’s practice cooperating with two certified breast cancer centers. Between 2016 and 2018, a total of *n* = 562 employed breast cancer patients who had received primary surgery were invited to participate in the study.

Patients were considered eligible if they were not older than 63 years, working at the time of diagnosis and had a diagnosis of invasive primary breast cancer [35]. Baseline data (t0) of *n* = 454 employed breast cancer patients were collected in the first weeks after primary surgery, corresponding to a response rate of 80.8%. The last follow-up (t3), which represents the data basis of this study, focused on the social participation of breast cancer patients on average five years after primary surgery. For this follow-up assessment, patients were sent a written questionnaire to their homes. A total of *n* = 372 women took part in this final survey, conducted between 2022 and 2023, corresponding to a response rate of 81.9% in relation to the initial sample (t0).

The sample characteristics are displayed in Table 1. The number of missing values for the included variables varied between *n* = 0 and *n* = 45, corresponding to 0–12% of cases. With the exception of the decision tree analysis, in which missing values are treated as a valid category, respondents with missing information were excluded.

**Table 1:** Sample characteristics of breast cancer survivors at t3 (*n* = 372)

	*n*	%
Age group		
20–49 yrs.	64	17.2
50–59 yrs.	188	50.5
60–69 yrs.	120	32.3
missing	0	
Level of schooling		
low (up to 9 yrs. of schooling)	46	12.8
intermediate (10 yrs. of schooling)	148	41.1
high (12 to13 yrs. of schooling)	166	46.1
missing	11	
Occupational status		
low/lower skilled	109	30.0
intermediate skilled	157	43.1
high skilled	98	26.9
missing	8	
Household net income		
low (up to 2249 €)	95	29.1
intermediate (2250 to 3999 €)	142	43.4
high (4000+ €)	90	27.5
missing	45	
Family status		
married	239	64.9
single	44	12.0
divorced, separated or widowed	85	23.1
missing	4	
Migration status ^1^		
yes	67	18.6
no	293	81.4
missing	12	
Labor force participation		
not employed^2^	112	31.8
part-time employed	131	37.2
full-time employed	109	31.0
missing	20	
Level of diagnosis-related complaints		
low (0–2 complaints)	105	28.9
intermediate (3–8 complaints)	150	41.2
high (> 8 complaints)	109	29.9
missing	8	
Mastectomy (t0)		
yes	84	22.8
no	284	77.2
missing	4	
Grading (t0)		
1	45	12.3
2	194	53.2
3	126	34.5
missing	7	
Tumor size (t0)		
≤ 2 cm	227	61.2
2 to 5 cm	117	31.5
> 5 cm	27	7.3
missing	1	
Chemotherapy (t0)		
yes	202	54.3
no	170	45.7
missing	0	

Notes ^1^ migration status = at least one parent was not born in Germany, ^2^ not employed = retired or unemployed and seeking for a job.

### Measures

#### Social factors

##### Socioeconomic status

We used the following variables to determine the socioeconomic status: *educational level*: low (up to 9 yrs. of schooling), intermediate (10 yrs. of schooling), high (12 to13 yrs. of schooling); *occupational status at t3*: low/lower status (unskilled, semi-skilled and skilled workers, farmers, salaried employees with simple tasks and civil servants in the ordinary service), intermediate skilled (self-employed persons without employees, salaried employees with qualified tasks and civil servants in the middle civil service), high skilled (self-employed persons with employees, salaried employees with highly qualified jobs, master craftsmen/master craftswomen, civil servants in the upper and higher levels of the civil service) and *household net income at t3*: low (up to 2249 €), intermediate (2250 to 3999 €) and high (4000+ €).

##### Sociodemographic variables

We analyzed the following sociodemographic variables: *age group* (20–49 yrs., 50–59 yrs. and 60–69 yrs.); *family status* (married, single and divorced, separated or widowed); *migration status*: at least one parent was not born in Germany (yes or no) and *labor force participation* (not employed/retired, part-time employed, full-time employed).

##### Participation in social activities

We defined social participation as “participation in social activities”, which was measured using a written questionnaire on 20 different leisure activities taken from the Socio-Economic Panel Study (GSOEP V.31). The GSOEP is a representative annual survey of German individuals aged 18 and older in private households, conducted by the German Institute for Economic Research [36]. The advantage of using this questionnaire is that it allows a comparison of the social activities of breast cancer patients with the general population as a control group. For each activity, participants were asked how often they do it in their leisure time. The answer categories were: (1) daily, (2) at least once a week, (3) at least once a month, (4) less often and (5) never. As social participation is defined as “a person’s involvement in social activities that provide social interactions within his/her community or society” [1, 2], we excluded those activities that are mainly carried out alone (e.g. reading books or newspapers, repairs to the house, flat or vehicles or gardening). A Principal Component analysis (PCA) was performed for the remaining variables in order to identify different dimensions of social participation. This analysis resulted in five dimensions of social participation: (1) sociocultural participation, (2) social participation in institutions, (3) social participation in the private sphere, (4) social participation via social media, and (5) passive leisure activities. Further information on this instrument and its validity can be found in Sperlich et al. [21]. For this study, we focused on three dimensions, with the following variables: *Sociocultural participation (4 items)*: (1) visits to cafes, pubs and restaurants; (2) visits to opera, theatre, classical concerts and exhibitions, (3) cinema visits and visits to pop and jazz concerts and clubs and (4) excursions and short trips. *Social participations in institutions (3 items)*: (1) participation in political parties, local politics and citizens’ initiatives; (2) voluntary activities in clubs and associations, social services, and (3) church visits and visits to religious events and *social participation in the private sphere (2 items)*: (1) reciprocal visits from neighbors, friends and acquaintances and (2) reciprocal visits from family members or relatives. We defined levels of sociocultural participation and social participation in institutions as ‘low’ if none of these activities were carried out at least monthly. Low levels of social participation in the private sphere were defined as reciprocal visits taking place less than once a week. Descriptive data on the dimensions of social participation can be found in Sperlich et al. [21].

##### Diagnosis-related complaints

Patients were given a list that included the following 22 breast cancer-related complaints: anxiety; depression; depressed moods; lack of drive/motivation; fatigue or constant tiredness; reduced physical resilience; weight loss or gain; dwelling in tissues or lymphedema; pain in joints, muscles or limbs; general psychological complaints; sleep disturbances; reduced resistance to psychological stress; restricted movement due to scarring; obstipation; hair loss; hot flushes; forgetfulness/poor concentration; impairment of sexuality; impaired fertility; nausea and vomiting; skin problems; damage to the peripheral nerves/neuropathy. Further information on the development of the diagnosis-related list of complaints and its validation can be found in Sperlich et al. [21]. For this study, we used the number of complaints classified into three categories as indicator of the extent of breast-cancer related symptoms: 0 to 2 complaints were classified as having a low number of complaints, 3 to 8 complaints as having a medium number and values of 9 or more complaints as having a high number of diagnosis-related complaints. The cut-off values were chosen so that the two groups with low and high complaints would be roughly the same size. 28.9% of the patients belonged to category ‘low complaints’ (0–2 symptoms), 41.1% to category ‘medium complaints’ (3–8 symptoms) and 30% to category ‘high complaints’ (9 or more symptoms).

### Statistical analyses

The association between social factors and the dimensions of social participation was analyzed by means of binomial regression analyses. Social participation serves as dependent variable and the socioeconomic and sociodemographic variables as independent variables, with a high socioeconomic status and sociodemographic characteristics assumed to be favorable as reference groups. We first conducted bivariate analyses for each of the variables, adjusted for age and estimated predicted probabilities with the post estimation command ‘margins’ that give adjusted prevalence. Prevalence ratios (PR) (age-adjusted) were calculated based on Poisson regression analyses. We used Poisson regression instead of logistic regression because the prevalence ratio is directly estimated here. Calculating the prevalence ratio is more suitable than calculating the odds ratio of logistic regression when the prevalence of the outcome is not rare (greater than 10%), as in this study [37]. Results are significant if the confidence intervals do not include the value 1 (p values ≤ 0.05). In addition to social determinants, we considered the number of diagnosis-related complaints divided into three categories (0–2; 3–8 and ≥ 9) as a further predictor of social participation. In order to determine whether diagnosis-related complaints are mediating the association between social factors and social participation, we additionally adjusted for the number of diagnosis-related complaints in each bivariate analysis.

Decision tree analysis was used to detect the socioeconomic and sociodemographic factors that are associated most strongly with restrictions in social participation. The decision tree approach identifies interactions within the independent variables, and displays a hierarchal illustration of their effects on the dependent variable. The order of independent variables in the tree are decided by its discriminating power, i.e. the independent variable that explains the most variance in the dependent variable is selected for the first split of the sample and the same approach is applied for each of the subsequent subgroups [38]. For interval-scaled variables, cut-off values are determined that best differentiate between the groups. It can happen that, depending on the specific subgroup, the same variable, such as the number of diagnosis-related complaints, has different cut-off values, for example for the low- and high-income group.

We used the statistical technique CHAID (Chi Squared Automatic Interaction Detection), first proposed by Kass [39]. A decision tree was modeled for each dimension of social participation (dependent variables), the independent variables included the number of diagnosis-related complaints and the social variables described above. CHAID makes the missing values a separate category and enables them to be used in tree building. In our analyses, the missing values did not appear as a separate category in the tree formation, but together with the valid values of the other variables (e.g. low income). In Figs. 2, 3 and 4, these values were labelled with a superscript 1 to indicate that the missing values were also assigned here. Chi-square values that are used to determine the splitting of nodes and merging of categories were calculated using the likelihood ratio method that is more robust than the Pearson method [40]. We set the significance level for splitting nodes and merging categories at *p* < 0.05 (alpha). The minimum number of cases required was *n* = 40 for the parent node and *n* = 20 for the child nodes. The maximum number of levels for child nodes was set to three. All statistical analyses were performed using the IBM SPSS Statistics 20.0 software package and STATA 13.

## Results

### Factors associated with low participation in social activities

**Fig. 1:**
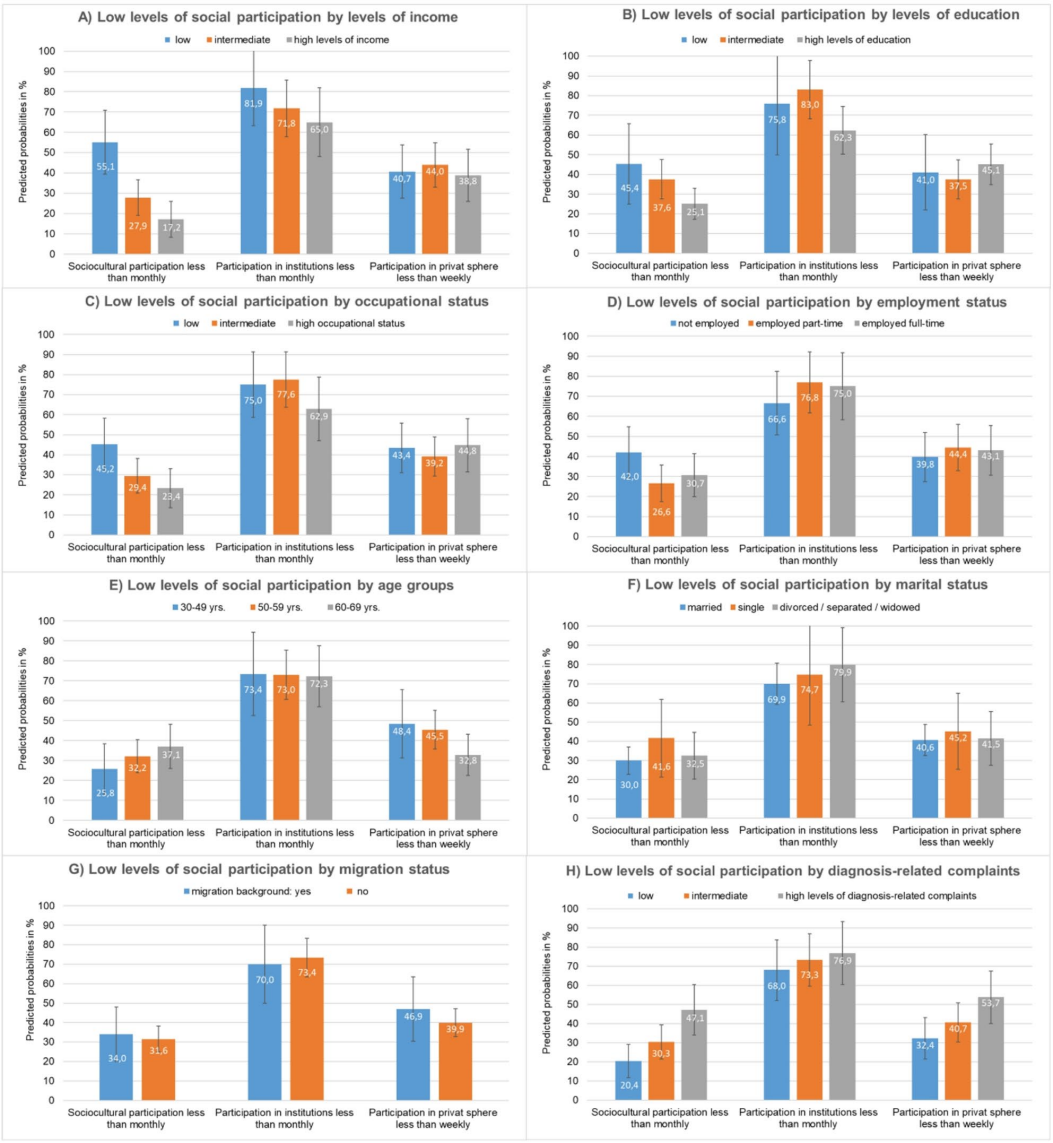
Adjusted prevalence (predicted probabilities) of social participation (three dimensions) by social factors (A-G) and diagnosis related complaints (H)

Low levels of *sociocultural participation* are significantly more frequent in breast cancer survivors with low household income (PR: 3.21, CI 1.77–5.80), low educational level (PR: 1.81, CI 1.04–3.15) and lower occupational status (PR: 1.93, CI 1.16–3.21) compared to survivors with high socioeconomic status (Table 2). As illustrated in Fig. 1, the proportion of low sociocultural participation increases with decreasing income, education and occupational status. For example, 25.1% of highly educated survivors show low levels of sociocultural participation while the respective proportion among those with intermediate and low education is 37.6% and 45.4% (Fig. 1B). Moreover, survivors with high levels of diagnosis-related complaints are more affected by low levels of sociocultural participation than those with low levels of complaints (PR: 2.31, CI 1.39–3.85) (Table 2; Fig. 1H). Low levels of sociocultural participation tended to increase with age, but this relationship was not statistically significant. All other socio-demographic factors, i.e. marital status, labor force participation and migration status, had no significant influence on socio-cultural participation.

**Table 2:** Prevalence ratio of low levels of social participation (three dimensions) for different social factors and diagnosis-related complaints in breast cancer survivors five years after first surgery (*n* = 372), adjusted for age

	Low levels of sociocultural participation		Low levels of participation in institutions		Low levels of participation in the private sphere	
	PR	95% CI	PR	95% CI	PR	95% CI
Age-groups						
20–49 yrs.	1		1		1	
50–59 yrs.	1.25	0.72; 2.17	0.99	0.71; 1.38	0.94	0.66; 1.22
60–69 yrs.	1.44	0.81; 2.55	0.98	0.69; 1.40	0.68	0.42; 1.08
Level of schooling						
high	1		1		1	
intermediate	1.50	0.99; 2.27	**1.33**	**1.02; 1.73**	0.83	0.59; 1.18
low	**1.81**	**1.04; 3.15**	1.22	0.82; 1.81	0.91	0.54; 1.53
Occupat. status						
high skilled	1		1		1	
intermediate skilled	1.26	0.75; 2.10	1.23	0.91; 1.68	0.87	0.59; 1.29
low/lower skilled	**1.93**	**1.16; 3.21**	1.19	0.85; 1.66	0.97	0.64; 1.46
Income						
high	1		1		1	
intermediate	1.62	0.89; 2.96	1.11	0.80; 1.53	1.13	0.75; 1.71
low	**3.21**	**1.77; 5.80**	1.26	0.89; 1.79	1.05	0.66; 167
Labor force participation						
full-time	1		1		1	
part-time	0.87	0.53; 1.41	1.02	0.76; 1.38	1.03	0.71; 1.51
not employed	1.37	0.85; 2.20	0.89	0.64; 1.24	0.92	0.60; 1.42
Marital Status						
married	1		1		1	
single	1.39	0.80; 2.39	1.07	0.73; 1.57	1.11	0.69; 1.80
Divorced/widowed^1^	1.08	0.70; 1.68	1.14	0.86; 1.52	1.02	0.69; 1.51
Migration status ^2^						
no	1		1		1	
yes	1.08	0.68; 1.48	0.95	0.70; 1.31	1.17	0.79; 1.75
Diagnosis-related complaints						
Low (0–2)	1		1		1	
Intermediate (3–8)	1.49	0.88;2.50	1.08	0.80; 1.46	1.26	0.83; 1.91
High (≥ 9)	**2.31**	**1.39;3.85**	1.13	0.82; 1.55	**1.66**	**1.09; 2.53**

^1^also including women who are separated from their partners, ^2^migration status = yes: at least one parent was not born in Germany, significant values (p values ≤ 0.05) highlighted in bold, *PR* Prevalence Ratio

With regard to *social participation in institutions*, the prevalence of low participation tends to be higher in patients with lower socioeconomic status. However, a significant higher prevalence is only found in women with intermediate levels of education compared to women with high levels of education (PR: 1.33, CI 1.02–1.73) (Table 2). Here too, age, marital status, labor force participation and migration status show no significant association with social participation in institutions.

The number of diagnosis-related complaints is the only variable that has a significant impact on *social participation in the private sphere*, in that survivors with a high number of complaints (≥ 9) were more affected by low participation levels compared to those with a low number (0–2). Survivors aged 60 to 69 years were less likely to be affected by low social participation in the private sphere compared to younger survivors aged 20 to 49 years (PR: 0.68, CI 0.42–1.08), but this association was not statistically significant. The other influencing factors also proved to be insignificant.

### The role of diagnosis-related complaints

Appendix Table 1 displays the effect of social factors on social participation controlled for the number of breast cancer-related complaints. It reveals that the effect size remained largely unchanged and this applies to all three dimensions of social participation.

### Subgroups of breast cancer survivors that are affected most by low levels of social participation

#### Sociocultural participation

**Fig. 2:**
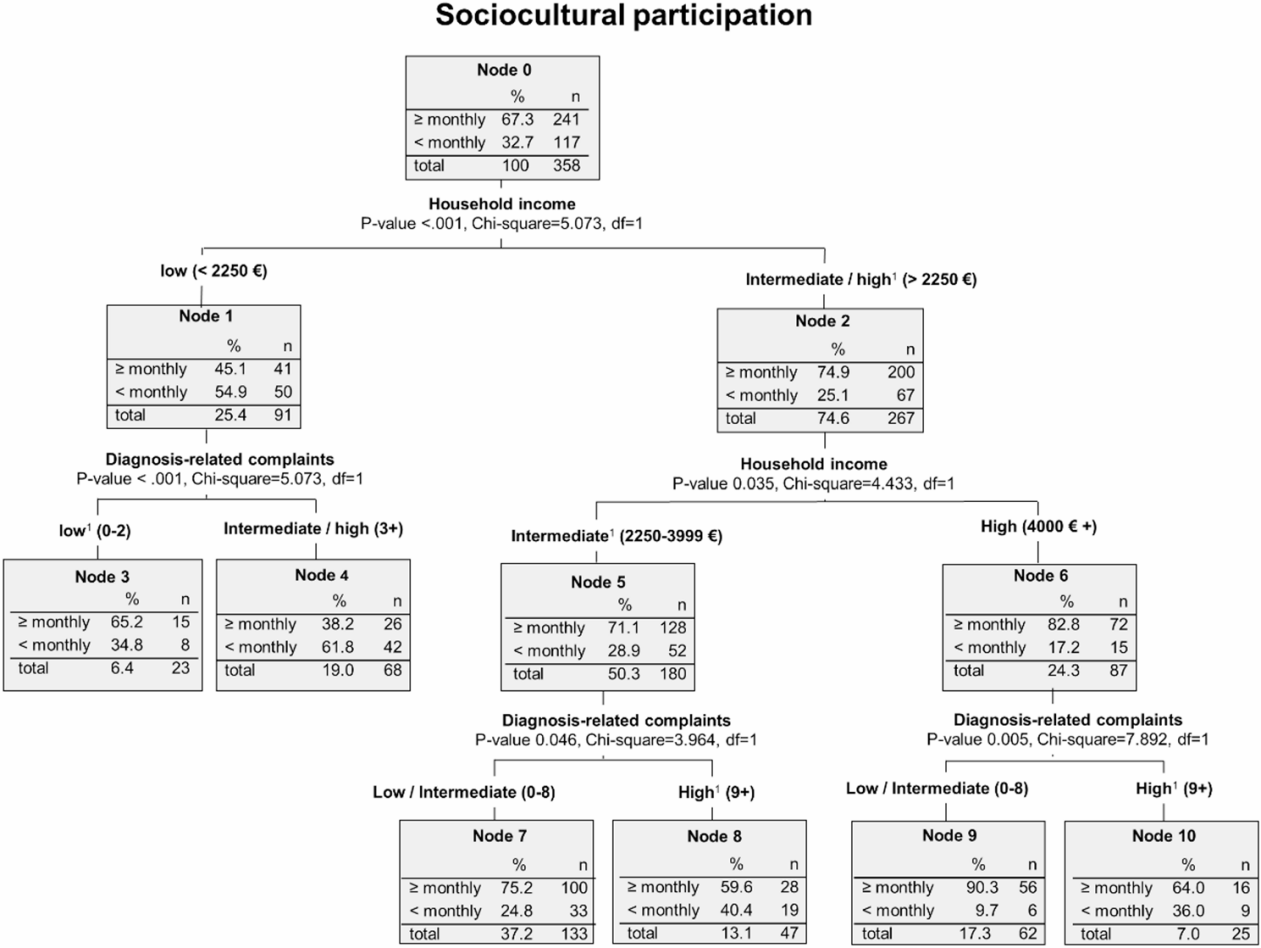
Subgroups of breast cancer survivors that are affected most by low levels of sociocultural participation - Decision tree analysis (CHAID method). Notes: ^1^ This category also includes the missing values.

Overall, 32.7% of breast cancer survivors have low levels of sociocultural participation, i.e. none of the activities in this dimension are performed at least monthly (Fig. 2, node 0). The first split of the tree, indicating the most important influencing factor on low sociocultural participation, is net household income. Among breast cancer survivors with low household income (< 2250 €), 54.9% show low levels of sociocultural participation (node 1) while the respective frequency of those with higher income is 25.1% (node 2). In node 2, the next split is between intermediate and high income. For patients of all income groups, the next most important influencing factor is the number of diagnosis-related complaints. Among patients with high income, the frequency of low sociocultural participation is further reduced to 9.7% if they do not have a high number of diagnosis-related complaints (node 9), that is nine and more complaints. By contrast, the highest frequency of low participation with 61.8% is found among low-income patients who have at least a medium number of diagnosis-related complaints (Fig. 2, node 4), that is three and more complaints.

#### Social participation in institutions

**Fig. 3:**
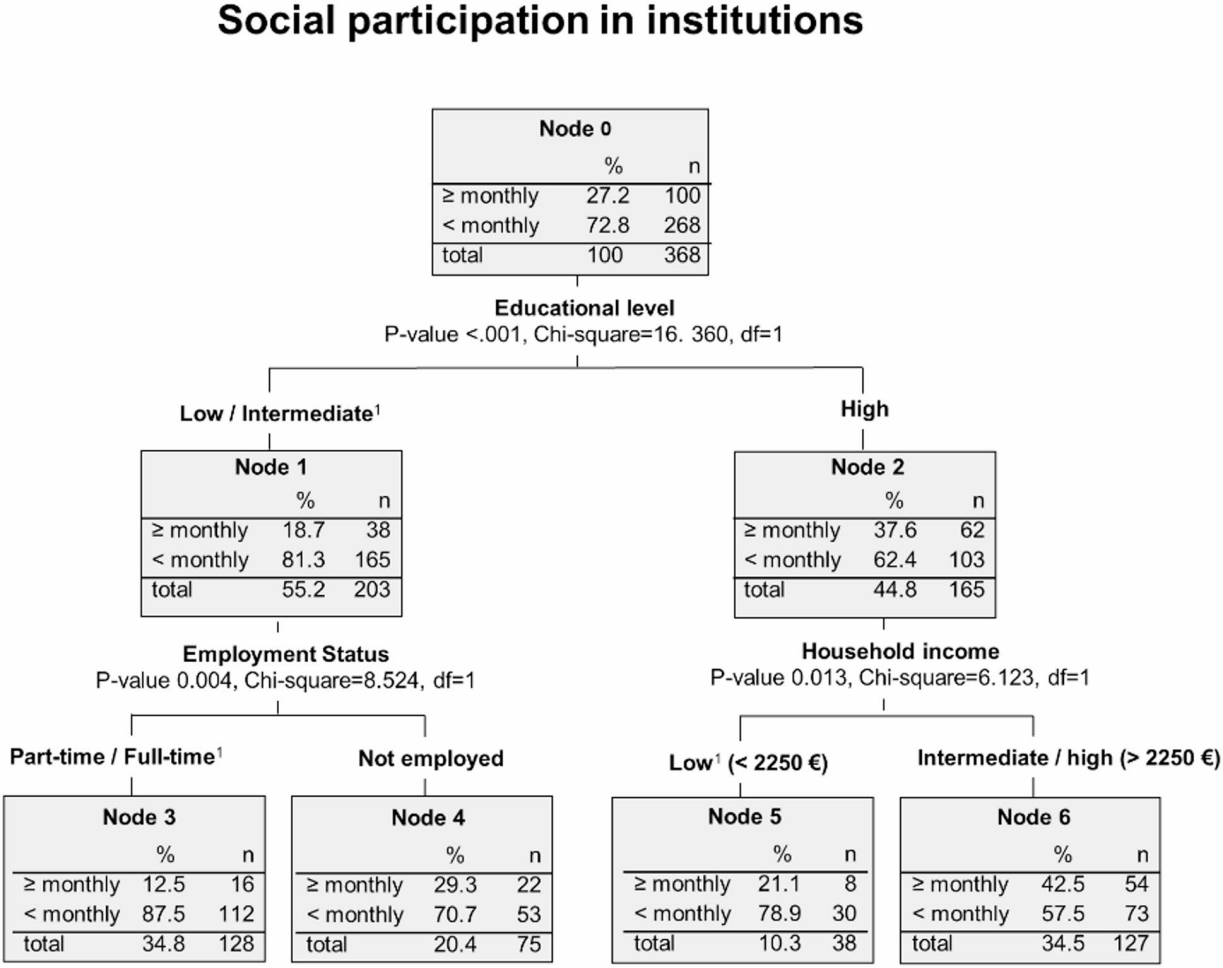
Subgroups of breast cancer survivors that are affected most by low levels of social participation in institutions - Decision tree analysis (CHAID method). Notes: ^1^ This category also includes the missing values.

Overall, 72.8% of the breast cancer survivors reported low levels of social participation in institutions, indicating no activity in this dimension at least once a month (Fig. 3, node 0). The most important factor influencing institutional participation is the level of education. While low levels of participation increased to 81.3% among patients with low levels of education (Fig. 3, node 1), the frequency is reduced to 62.4% among those with higher levels of education (Fig. 3, node 2). Survivors with the combination of higher educational level and a household income > 2250 € show the lowest frequency of low participation (57.5%) (Fig. 3, node 6). By contrast, the combination of lower educational level and being employed (part-time or full-time) is associated with the highest frequency of low social participation in institutions (87.5%, Fig. 3, node 3).

#### Social participation in the private sphere

**Fig. 4:**
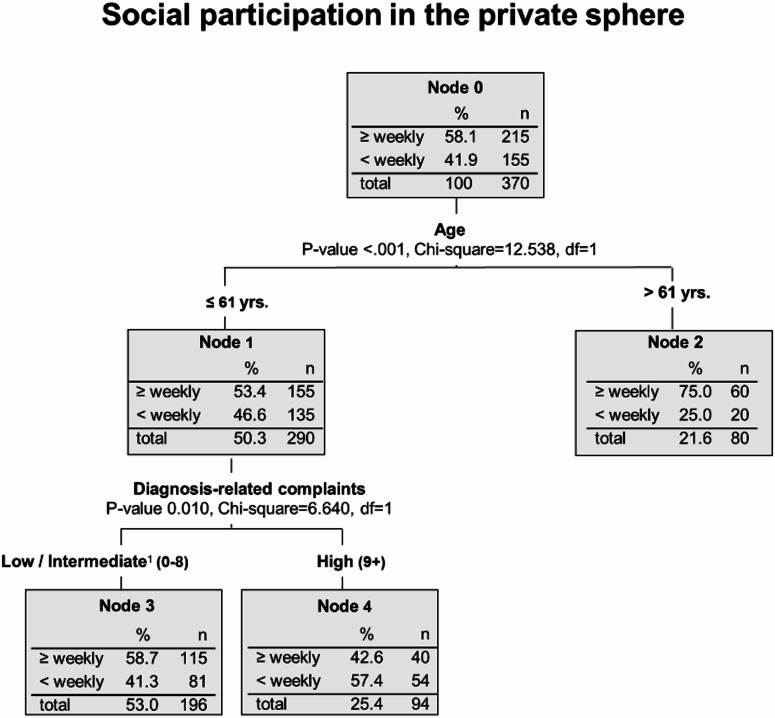
Subgroups of breast cancer survivors that are affected most by low levels of social participation in the private sphere - Decision tree analysis (CHAID method). Notes:^1^ This category also includes the missing values.

Of all the participants included, 41.9% do not receive visits from friends or family members at least once a week, nor do they visit them (Fig. 4, node 0). The most important influencing factor on low levels of social participation in the private sphere is age. In patients aged 62 and older, the proportion of low social participation drops to 25.0% (Fig. 4, node 2), while in those younger than 62, it rises to 46.6% (Fig. 4, node 1). The lowest levels of social participation are found among patients aged below 62 years and who have a high number of diagnosis-related complaints. In this node, the frequency increased to 57.4% (Fig. 4, node 4).

## Discussion

In this study, we analyzed the influence of sociodemographic and socioeconomic factors on social activities among breast cancer survivors on average five years after first surgery. We found no significant associations between social participation and marital status, labor force participation or migration status. By contrast, we found clear socioeconomic inequalities in the sociocultural participation of breast cancer survivors.

### Social inequalities in social participation among breast cancer survivors

Studies that focus on social inequalities in social activities outside of work are relatively rare. Of the few studies, Zhu et al. [3] found no significant effect of school education or income level on social participation in breast cancer patients. Our study found that, in particular, low sociocultural participation is more common among socially disadvantaged breast cancer survivors. This applies to all three indicators of socioeconomic status, i.e. educational level, occupational status and household income. Our results indicate that socially disadvantaged breast cancer survivors are less likely to participate in social life, e.g. visiting cafés, pubs or restaurants, making excursions or short trips or join cultural events. We also found that there are social inequalities in terms of social participation in institutions, i.e. participation in citizens’ initiatives, political parties, voluntary work or church attendance was higher among high educated breast cancer survivors. Our key finding that socioeconomically disadvantaged women are less involved in social activities could be an additional explanation for their poorer prognosis compared to women with higher socioeconomic status, as found in previous studies [41–43].

### The role of diagnosis-related complaints

After controlling for breast cancer-related complaints, effects of socioeconomic status on social participation remained largely stable. This indicates that diagnosis-related complaints are not responsible for the association between socioeconomic disadvantages and restrictions in social participation among breast cancer survivors. Rather, our results indicate that both factors, low income and cancer-related complaints, are independently associated with greater restrictions on sociocultural participation. Previous studies indicate that older adults with low socioeconomic status are more likely to have restrictions in social participation compared to those who are better of [7, 25–28]. Therefore, it can also be assumed for our study that socially disadvantaged women already had fewer opportunities for sociocultural participation due to financial constraints before their diagnosis. However, it should also be noted that a breast cancer diagnosis and related health issues could have led to a reduction in or cessation of employment, which could have resulted in a deterioration of the financial situation.

Remarkably, only 9.7% of breast cancer survivors with a high household income reported restrictions in their sociocultural participation, even if they had up to eight diagnosis-related complaints. In contrast, for low-income patients, more than two complaints were associated with restrictions in sociocultural participation, which applied to 61.8% in this group. This suggests that complaints related to diagnosis have a greater impact in women with low income and that they are more vulnerable in this regard. However, it is also possible that complaints are more severe in socially disadvantaged women than in those who are better off, leading to a stronger negative effect on social participation. Additional information about the severity of complaints would provide more insight into this issue, which should be the subject of further investigation. Regardless of which explanation applies, it can be assumed that diagnosis-related complaints may further exacerbate the income-related restrictions on social participation. Women with low income, who continue to have complaints, are therefore a particularly vulnerable group in terms of low sociocultural participation, which requires special attention in follow-up care.

### Different significance of social factors depending on the type of social participation

In contrast to sociocultural participation, only a moderate effect of socioeconomic factors was found for social participation in institutions and no association at all could be established for social participation in the private sphere that relates to mutual visits by friends or family. This suggests that, in particular, reciprocal visits by friends and relatives, which take place at home, are apparently less influenced by financial resources and level of education. Rather, younger survivors (< 62 years) with a high number of breast cancer-related complaints were more affected by restrictions in this social activity. Our findings indicate that the importance of social factors for social participation cannot be generalized, but may vary depending on the aspect under consideration.

Moreover, it was found that the three socioeconomic factors have different significance depending on the respective dimension of social participation. While a lower level of education was primarily associated with lower social engagement in institutions, a low household income proved to be most important for restrictions on sociocultural participation. Socio-epidemiological studies have shown that the respective influence of the three socioeconomic indicators on the risk of disease can vary and that a differentiated consideration of all three indicators is therefore indicated [44]. The present results suggest that socioeconomic factors may also have differential significance for the course of disease and coping with illness, which points to the importance of using all three indicators when analyzing the course of the disease, if possible.

### Strengths and limitations

The strengths of this study include the longitudinal and multicenter study design and the high response rate of 81.9% for this third follow up in relation to the first sample (t0). However, we also acknowledged relevant study limitations. Firstly, the study population bases on breast cancer patients who were employed at t0. Thus, our findings cannot claim to be representative for all breast cancer patients. In addition, we used the net household income not adjusted for the number of household members which provides a more accurate picture of the financial resources of the household. This was because we assessed income as a categorical variable with predefined income groups (e.g. 2250 to 2999 €) for which the adjusted net income cannot be determined precisely. However, as part of sensitivity analyses, we have used the respective mean value of the income groups (e.g. 2650 € for the group 2250–2999 €) as an approximation of the average incomes and calculated the household-adjusted net equivalent income based on these values. The use of this adjusted income variable yielded largely identical findings and confirmed the central importance of income for restrictions particularly in sociocultural participation. Moreover, the focus of our study was on the influence of social factors, while the significance of diagnosis-related complaints was only measured with the number of complaints. Further analyses should therefore explore in more depth the complexity of social factors interacting with specific disease related complaints helping to understand barriers of social participation in breast cancer survivors.

### Practical implications

In contrast to unavoidable biological and genetic risk factors, social participation is a modifiable health determinant. The potential consequences of limited social participation in breast cancer patients include poor functional recovery, well-being and health-related quality of life [8, 9, 11]. Moreover, Glass et al. found that socially active older adults also had a lower risk of overall mortality than those who were less socially active. They concluded that social activities offer survival benefits equivalent to those of physical activity [10]. Given the importance of social participation, a regular assessment of social participation and its barriers and facilitators should be carried out throughout therapy, including follow-up. The present study showed that socially disadvantaged breast cancer survivors experience greater restrictions in social participation five years after first surgery. Therefore, these women should be given special attention by all professional groups involved in treatment and therapy to promote the social participation. Psycho-oncology, which aims to help patients to cope with their illness and any psychological, social and functional health problems that may arise, plays an important role in this regard. In addition, cancer self-help groups play a vital role in improving patients’ social participation, as they have been shown to improve emotional functioning, coping skills, quality of life and personal relationships [45]. Participation in a support group itself represents a social activity that patients should be encouraged to take part by healthcare professionals.

## Conclusion

We found evidence for socioeconomic inequalities in the participation in social activities of breast cancer survivors to the detriment of those with low socioeconomic status. In particular, women with low income who continue to suffer from breast cancer-related complaints were less involved in sociocultural activities even five years following first surgery compared to those who are socioeconomically better off. Our results show that a comprehensive assessment of social participation and early intervention are needed to prevent long-term limitations in social participation, especially in socially disadvantaged breast cancer survivors.

## Supplementary Information

Supplementary Material 1.

Supplementary Material 2.

## Data Availability

The data that support the findings of this study are available on request from the corresponding author. The data are not publicly available due to privacy restrictions.
