# Supplementary material for: Breast cancer survivors’ participation in social activities five years after primary surgery – are there social inequalities?
Source: BMC Cancer. 2025 Nov 27;25:1829. doi: 10.1186/s12885-025-15297-0 (PMC12659312; doi:10.1186/s12885-025-15297-0)
Supplement: Supplementary file 1 — Supplementary Material 1. [file 12885_2025_15297_MOESM1_ESM.docx]

**Questionnaire**

***With this questionnaire, we would like to find out how you have been doing since the last survey 3-5 years ago.***

### Social Factors Multiple choice possible, please check for each of the options whether it applies to you!

Please also complete this form if you are currently on sick leave as an employed person***.***

I am...

| Employed (dependent) | O |
| --- | --- |
| Employed (self-employed) | O |
| In process of gradual reintegration | O |
| In a measure organized by the Federal Employment Agency | O |
| Retired with paid secondary employment | O |
| Retired without paid secondary employment Since month____ year______ | O |
| In partial retirement with zero working hours or in the release phase | O |
| Unemployed | O |
| other, namely:__________________________________________________ | O |

### How much time do you actually work per week on average, including any overtime?

### *Please also answer this question if you are currently on sick leave.*

|  |  | , |  | hours per week |  | does not apply O |
| --- | --- | --- | --- | --- | --- | --- |

### What is your marital status?

| Married and living with my spouse | O |
| --- | --- |
| Married and living with my spouse or with my registered partner | O |
| Married and living separately from my spouse | O |
| Married and living separately from my spouse or from my registered partner | O |
| Single | O |
| Divorced | O |
| Widowed | O |

### What is the total net monthly income of your household?

*This includes the wages or income of all persons living and working in the household, as well as rental income, child benefit, parental allowance, unemployment benefit, Hartz IV, income support, accommodation costs, housing benefit, pensions, etc.*

| Below 750 € | 750 to under 1250 € | 1250 to under 1750 € | 1750 to under 2250 € | 2250 to under 3000 € | 3000 to under 4000 € | |
| --- | --- | --- | --- | --- | --- | --- |
| O | O | O | O | O | O | |
| 4000 to under 5000 € | 5000 € and more | I don’t want to answer | I don’t know |  |  | |
| O | O | O | O |  |  |  |

### Breast cancer-related complaints

### Multiple choice possible, please check for each of the options whether it applies to you!

### If you have no complaints, please check “none”.

| Fatigue (constant tiredness) | O |  | Skin problems | O |
| --- | --- | --- | --- | --- |
| Sleep disorders | O |  | Hot flushes | O |
| Forgetfulness, poor concentration, word-finding difficulties, short attention span | O |  | Reduced resistance to mental stress | O |
|  |  |  | Lack of drive / motivation | O |
| Reduced physical resilience | O |  | General mental complaints | O |
| Nausea and vomiting | O |  | Depressed moods | O |
| Damage to the peripheral nerves (neuropathy) | O |  | Depression | O |
| Obstipation | O |  | Anxiety disorders | O |
| Swelling in the tissue due to blocked lymph fluid (lymphedema) | O |  | Impaired fertility | O |
| Hair loss | O |  | impairment of sexuality | O |
| pain in joints, muscles or limbs | O |  | restricted movement due to scarring | O |
| weight loss or gain | O |  | Others, namely: ________________________________  ______________________________ | O |
| **none** | O |  |  |  |

### Social participation in leisure activities

### The following section contains questions about your leisure activities.

**Please indicate how often you do each activity: daily, at least once a week, at least once a month, less often or never?**

|  |  | daily | at least once a week | at least once a month | less often | never |
| --- | --- | --- | --- | --- | --- | --- |
| 1 | Eating or drinking out  (café, bar, restaurant) | O | O | O | O | O |
| 2 | Reciprocal visits from neighbors, friends or acquaintances | O | O | O | O | O |
| 3 | Reciprocal visits from family members or relatives | O | O | O | O | O |
| 4 | Contact with friends or relatives abroad (including by phone, email, internet telephony) | O | O | O | O | O |
| 5 | Use of social online networks / chat services (e.g. Facebook, Instagram, Twitter, WhatsApp) | O | O | O | O | O |
| 6 | Excursions and short trips | O | O | O | O | O |
| 7 | Participation in parties, local politics, citizens' initiatives | O | O | O | O | O |
| 8 | Voluntary activities in clubs, associations or social services | O | O | O | O | O |
| 9 | Going to church, attending religious events | O | O | O | O | O |
| 10 | Watching TV, movies, series or videos (including media libraries, internet streams / DVDs, etc.) | O | O | O | O | O |
| 11 | Playing computer, online, console or smartphone games | O | O | O | O | O |
| 12 | Reading books (including e-books) | O | O | O | O | O |
| 13 | Reading (daily) newspapers (including e-papers) | O | O | O | O | O |
| 14 | Artistic and musical activities (painting, making music, photography, theater, dance) | O | O | O | O | O |
| 15 | Home repairs, repairs to the apartment or vehicles, gardening, vehicle maintenance | O | O | O | O | O |
| 16 | Active sporting activities | O | O | O | O | O |
| 17 | Attending sporting events | O | O | O | O | O |
| 18 | Going to the cinema, attending pop or jazz concerts, dance events, disco | O | O | O | O | O |
| 19 | Attending events such as opera, classical concerts, theater, exhibitions | O | O | O | O | O |
| 20 | Just do nothing, hang around, dream | O | O | O | O | O |
