# Supplementary material for: Breast cancer survivors’ participation in social activities five years after primary surgery – are there social inequalities?
Source: BMC Cancer. 2025 Nov 27;25:1829. doi: 10.1186/s12885-025-15297-0 (PMC12659312; doi:10.1186/s12885-025-15297-0)
Supplement: Supplementary file 2 — Supplementary Material 2. [file 12885_2025_15297_MOESM2_ESM.docx]

**Appendix Table 1:** Prevalence ratio (PR) of low levels of social participation (three dimensions) for different social factors in breast cancer survivors five years after first surgery (n=372), Germany, adjusted for age and diagnosis-related complaints

|  | **Low levels  of sociocultural participation** | | **Low levels  of participation in institutions** | | **Low levels of  participation in the private sphere** | |
| --- | --- | --- | --- | --- | --- | --- |
|  | **PR** | **95% CI** | **PR** | **95% CI** | **PR** | **95% CI** |
| **Age-groups** |  |  |  |  |  |  |
| 20-49 yrs. | 1 |  | 1 |  | 1 |  |
| 50-59 yrs. | 1.19 | 0.67; 2.11 | 1.00 | 0.71; 1.40 | 0.88 | 0.61; 1.14 |
| 60-69 yrs. | 1.57 | 0.87; 2.84 | 1.01 | 0.70; 1.45 | 0.69 | 0.43; 1.10 |
| **Household income** |  |  |  |  |  |  |
| high | 1 |  | 1 |  | 1 |  |
| intermediate | 1.66 | 0.90; 3.07 | 1.11 | 0.80; 1.55 | 1.08 | 0.74; 1.43 |
| low | **3.05** | **1.65; 5.66** | 1.25 | 0.87; 1.81 | 0.91 | 0.55; 1.26 |
| **Level of schooling** |  |  |  |  |  |  |
| high | 1 |  | 1 |  | 1 |  |
| intermediate | 1.42 | 0.94; 2.16 | **1.32** | **1.01; 1.72** | 0.79 | 0.56; 1.13 |
| low | **1.76** | **1.00; 3.10** | 1.20 | 0.80; 1.80 | 0.94 | 0.56; 1.60 |
| **Occupational status** |  |  |  |  |  |  |
| high skilled | 1 |  | 1 |  | 1 |  |
| intermediate skilled | 1.30 | 0.77; 2.19 | 1.25 | 0.92; 1.71 | 0.84 | 0.57; 1.24 |
| low / lower skilled | **1.95** | **1.16; 3.28** | 1.21 | 0.92; 1.71 | 0.98 | 0.65; 1.47 |
| **Employment status** |  |  |  |  |  |  |
| full-time | 1 |  | 1 |  | 1 |  |
| part-time | 0.89 | 0.54; 1.46 | 1.03 | 0.63; 1.24 | 1.06 | 0.72; 1.55 |
| not employed | 1.31 | 0.81; 2.10 | 0.89 | 0.72; 1.04 | 0.89 | 0.58; 1.37 |
| **Marital Status** |  |  |  |  |  |  |
| married | 1 |  | 1 |  | 1 |  |
| single | 1.38 | 0.79; 2.39 | 1.06 | 0.72; 1.56 | 1.02 | 0.62; 1.67 |
| divorced/widowed^1^ | 1.03 | 0.66; 1.61 | 1.15 | 0.86; 1.53 | 0.98 | 0.66; 1.45 |
| **Migration status**^2^ |  |  |  |  |  |  |
| no | 1 |  | 1 |  | 1 |  |
| yes | 1.04 | 0.65; 1.66 | 0.94 | 0.68; 1.30 | 1.19 | 0.80; 1.78 |

Notes: PR = Prevalence Ratio, ^1^also including women who are separated from their partners, ^2^migration status = yes: at least one parent
was not born in Germany, significant values (p values ≤0.05) highlighted in bold
